# Antioxidant and Antifungal Effects of *Lavandula stoechas* Aqueous Extract Against *Aspergillus niger* and *Fusarium oxysporum* as a Potential Natural Preservative and Crop Protection Agent in the Agrifood Sector

**DOI:** 10.1002/cbdv.71190

**Published:** 2026-04-09

**Authors:** Imane Namoune, Fairouz Boubrik, Rima Yakoubi, Lynda Gali, Djamila Benouchenne, Samira Bendjedid, Hadj Sadok Tahar, Salim Terriche, Hamdi Bendif, Anis Ahmad Chaudhary, Tarek H. Taha, Walid Elfalleh, Stefania Garzoli

**Affiliations:** ^1^ Laboratory of Applied Biochemistry, Department of Biotechnology, Faculty of Nature and Life Sciences Ferhat Abbas University of Setif 1 Setif Algeria; ^2^ Laboratory of Characterization and Valorization of Natural Resources (L.C.V.R), Faculty of Life and Natural Sciences and of Earth and Universe Sciences Mohamed El Bachir El Ibrahimi University Bordj Bou Arreridj Algeria; ^3^ Plant and Animal Production Improvement and Development Laboratory, Department of Agronomic Sciences, Faculty of Nature and Life Sciences Ferhat Abbas University of Setif 1 Setif Algeria; ^4^ Laboratory of Biotechnology, Environment and Health, Faculty of Nature and Life Sciences Blida 1 University Blida Algeria; ^5^ Biotechnology Research Center—CRBt Constantine Algeria; ^6^ Laboratoire De Génétique, Biochimie Et Biotechnologie végétale, Faculté Des Sciences de La Nature et De La Vie Université Des Frères Mentouri Constantine 1 Constantine Algeria; ^7^ Higher National School of Biotechnology Taoufik KHAZNADAR Nouveau Pôle Universitaire Ali Mendjli Constantine Algeria; ^8^ Laboratoire De Biologie, Eau et Environnement (LBEE), Faculté Des Sciences de La Nature et De La Vie Et Sciences De La Terre et De l'Univers. Université 8 Mai 1945 Algérie; ^9^ Laboratoire Des Sciences de L'environnement Et D'agro‐Ecologie (SEAE), Faculté Des Sciences de La Nature et De La Vie, Département De Biologie Université Chadli Bendjedid El Tarf Algérie; ^10^ Department of Veterinary Sciences Constantine 1 University El‐Khroub Algeria; ^11^ Department of Biology, College of Science Imam Mohammad Ibn Saud Islamic University (IMSIU) Riyadh Saudi Arabia; ^12^ Department of Chemistry and Technologies of Drug Sapienza University, P. Le Aldo Moro Rome Italy

**Keywords:** antifungal activity, antioxidant activity, aqueous extract, food preservation, HPLC analysis*s*, *Lavandula stoechas*, phenolic compounds

## Abstract

The antioxidant and antifungal activities of (*Lavandula stoechas) L. stoechas* aqueous extract were studied to examine their potential application against food and crop spoilage. Total phenolic (TPC) and flavonoids (TFC) contents were quantified and individual polyphenols were analyzed by high‐performance liquid chromatography (HPLC). In vitro antioxidant assays including DPPH, ABTS, and Galvinoxyl radical scavenging, β‐carotene bleaching assay, reducing power, CUPRAC, and phenanthroline were applied. The antifungal effect of the extract was evaluated against the two plant pathogenic fungi *Aspergillus niger* and *Fusarium oxysporum* using the agar diffusion method. High TPC (197.23 ± 0.12 µg GAE/mg) and TFC (166.93 ± 1.15 µg QE/mg) contents were recorded with the extract. In total, 36 compounds were identified by HPLC, of which 35 are phenolic compounds, with rosmarinic acid (25.2%), *p*‐coumaric acid (7.7%) and luteolin‐7‐*O*‐glucoside (7.6%) being the representative phenolic compounds. *L. stoechas* demonstrated strong antioxidant activity in all the methods used. The extract completely inhibited (100% inhibition) the growth of *A. niger* at a concentration of 5%, while at the same concentration the extract inhibited *F. oxysporum* with a percentage of 89.62%. These preliminary results suggest the possible use of *L. stoechas* extract as a potential alternative for preventing plant diseases, reducing post‐harvest losses and for food preservation.

## Graphical abstract

Phytochemical Investigation and Antioxidant and Antifungal Effects of *Lavandula stoechas* Aqueous Extract.

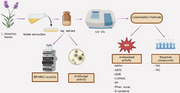

## 1 Introduction

Ensuring food security while respecting the environment within the concept of sustainable agriculture is the main challenge of the agri‐food sector. Fighting plant diseases and reducing post‐harvest losses are the main strategies to increase the production and prevent shortages. Fungal rot is among the main causes of fruit and vegetable spoilage after harvest, due to a favorable environment, including low pH, high humidity, and a composition that promotes the growth of these microorganisms [1]. Many fungal species, such as *Penicillium* spp., *Fusarium* spp., *Aspergillus* spp., and *Colletotrichum* spp., have been identified as the main agents responsible for the deterioration of fruits and vegetables after harvest, in addition to their ability to produce mycotoxins with potentially carcinogenic and nephrotoxic actions [2]. Synthetic chemicals, including pesticides, herbicides, insecticides, and fungicides, and so on are commonly used in agriculture to combat harmful biotic agents and other factors that induce plant diseases and postharvest deterioration of crops. However, the excessive use of these chemicals over time has led to serious consequences for the ecosystem as well as impacts on human health following prolonged exposure [3, 4].

Besides, quality preservation for the final foodstuff ready for consumption represents another challenge for manufacturers in the food industry. In this context, lipid oxidation constitutes a major factor involved in the degradation of the nutritional and organoleptic qualities of foodstuffs during their processing and storage. Synthetic antioxidants are commonly added to foods to counteract oxidation reactions, thus preventing rapid deterioration while preserving their qualities. Although effective, scientific reports have highlighted the harmful effects of prolonged consumption of these synthetic preservatives, including cytotoxic and genotoxic effects [5, 6]. Consequently, natural bioactive compounds from plant secondary metabolism, such as essential oils and polyphenols, have been the subject of several studies aimed at applying them as alternatives against phytopathogenic agents and preventing losses in the agri‐food sector [7, 8] while minimizing the hazardous impacts of chemicals and synthetic additives [9].

The flora of the Mediterranean region is characterized by great diversity, including medicinal and culinary herbs that have long been employed in cooking and folk medicine due to their aromatic and healing properties*. L. stoechas*, from the Lamiaceae family, is renowned for its medicinal attributes, particularly valued for its tonic and carminative effects and was used to treat inflammatory disorders and lung infections. Data from the literature have already described the antifungal, antioxidant, antibacterial, insecticidal, herbicidal and anti‐inflammatory actions of its essential oil, extracts and isolated molecules [10, 11, 12, 13, 14, 15, 16].

The potential application of *L. stoechas* essential oil in agriculture is most often mentioned, and although data are available on the pharmacological effects of phenolic extracts from this plant, their applications in crop protection and post‐harvest crop preservation remains largely unexplored. Besides, most studies on the extraction of polyphenols from plant materials have reported the use of organic solvents. Concerns related to the safety, sustainability, and regulatory restrictions associated with these solvents in the food industry have spurred increasing interest in the use of alternative and environmentally friendly extraction methods [17]. The main objective of this study is to examine the antioxidant and antifungal effects of the aqueous extract of *L. stoechas*, with a view to its application in food preservation, plant and crop protection against phytopathogenic agents. The composition of the extract was also determined by chromatography (HPLC) in order to better understand and interpret the results obtained for the biological effects.

## 2 Material and Methods

### 2.1 Plant Material

The plant material of *L. stoechas* collected in Algeria was verified for its identity by comparison with a reference herbarium specimen authenticated by botanists, with a voucher deposited in the Herbarium of the Higher Normal School of Kouba (HNIA) under the accession code HNIA = FA = N: P69. Leaves of *L. stoechas* were harvested in April 2018 from Djbal Guerbes Sanhadja, Skikda (north‐eastern Algeria; 36°45′ N, 7°13′ E). The collected material was washed, air‐dried at room temperature for two weeks, and subsequently ground into a fine powder using a laboratory mill.

### 2.2 Preparation of Aqueous Extract

The aqueous extract of *L. stoechas* was obtained according to the method reported by Yakoubi et al. [18]. Briefly, 100 g of the dried plant powder was macerated in 500 mL of distilled water for 24 h. The mixture was then filtered, and the filtrate was freeze‐dried to yield the aqueous extract (11.22 g), which was stored at 4°C until further use.

### 2.3 Quantification and Identification of Polyphenols

#### 2.3.1 Determination of Total Phenolic Content (TPC)

Total phenolic content (TPC) was quantified spectrophotometrically using the Folin–Ciocalteu reagent (FCR) method as originally described by Singleton and Rossi [19], following the protocol of Müller et al. [20]. In a 96‐well microplate, 20 µL of the extract was combined with 100 µL of FCR diluted 1:10 with distilled water and 75 µL of sodium carbonate solution (7.5%). The reaction mixture was incubated in the dark at room temperature for 2 h. Absorbance was then recorded at 765 nm using a microplate reader (PerkinElmer EnSpire, Singapore). TPC values were expressed as micrograms of gallic acid equivalents per milligram of extract (µg GAE/mg).

#### 2.3.2 Determination of Total Flavonoids Content (TFC)

Total flavonoid content (TFC) was determined according to the method described by Topçu et al. [21]. Briefly, 50 µL of the extract solution (1 mg/mL) was dispensed into a microplate well, followed by the addition of 130 µL of methanol, 10 µL of potassium acetate (1 M), and 10 µL of aluminum nitrate (10%). The reaction mixture was incubated at room temperature for 40 min, after which absorbance was measured at 415 nm. A calibration curve was constructed using quercetin as the standard, and TFC was expressed as micrograms of quercetin equivalents per milligram of extract (µg QE/mg).

#### 2.3.3 RP‐HPLC‐UV–Visible Analysis of Phenolics Compounds

Polyphenolic profiling was performed by reversed‐phase high‐performance liquid chromatography (RP‐HPLC) using an Agilent 1260 Infinity HPLC system (Agilent Technologies, Santa Clara, CA, USA) equipped with a quaternary pump, autosampler, thermostatted column compartment, and UV–vis diode array detector (DAD). Chromatographic separation was achieved on a C18 reversed‐phase column (150 × 4.6 mm, 5 µm particle size) protected by a guard column of the same stationary phase. The mobile phase consisted of solvent A (water acidified with 1% acetic acid, v/v) and solvent B (HPLC‐grade methanol). All solvents were filtered through a 0.45 µm membrane filter and degassed by ultrasonication prior to use. The gradient elution program was as follows: 20% B (0–5 min), 20%–50% B (5–20 min), 50%–80% B (20–30 min), and 80%–20% B (30–35 min), followed by re‐equilibration at 20% B (35–40 min). The overall analytical cycle was 100 min to ensure complete column stabilization between runs. The flow rate was maintained at 1.0 mL/min, the injection volume was 20 µL, and the column temperature was set at 35°C to ensure reproducibility. Detection was carried out at 254 and 280 nm, wavelengths suitable for the absorption of phenolic acids and flavonoids. Identification of polyphenolic compounds was based on their retention times in comparison with reference standards. Quantification was performed using external calibration curves constructed from standard solutions at different concentrations. The method was validated in terms of linearity, precision, and sensitivity, with acceptable correlation coefficients (R^2^), and limits of detection (LOD) and quantification (LOQ) determined according to standard analytical procedures.

### 2.4 Antioxidant Activity Assays

All tests used for evaluating in vitro antioxidant activity were performed on a 96‐well microplate and absorbance was recorded using a multimode microplate reader (PerkinElmer EnSpire 2300, Singapore).

#### 2.4.1 DPPH Radical Scavenging Assay

The free radical scavenging activity was evaluated using the DPPH assay as described by Blois [22]. Briefly, 40 µL of the extract at different concentrations was added to a microplate well containing 160 µL of a 0.1 mM methanolic DPPH solution. The reaction mixture was incubated for 30 min at room temperature in the dark, after which absorbance was measured at 517 nm. Butylated hydroxytoluene (BHT) and butylated hydroxyanisole (BHA) were used as reference antioxidants. The percentage of DPPH radical inhibition was calculated using the following equation:

(1) $$I\left(\%\right)=[\left(A_{c}-A_{s}\right)/A_{c}]\times 100$$

where *I* represents the percentage of inhibition, *A*_c_ represents absorbance of the control, and *A*_s_ represents absorbance of the reaction containing the sample at different concentrations.

The concentration of the sample reducing the initial absorbance of the DPPH solution by 50%, called the IC_50_, was determined from the curve of the percentages of inhibition obtained at different concentrations.

#### 2.4.2 ABTS Radical Scavenging Assay

The ABTS radical scavenging activity was evaluated according to the method described by Re et al. [23]. The ABTS•^+^ stock solution was generated by reacting 7 mM ABTS with 2.45 mM potassium persulfate in aqueous medium for 12–16 h at room temperature in the dark. The resulting radical cation solution was subsequently diluted with distilled water to obtain an absorbance of 0.70 ± 0.02 at 734 nm. Antioxidant activity was assessed by mixing 160 µL of the diluted ABTS•^+^ solution with 40 µL of the extract. After 10 min of incubation, absorbance was measured at 734 nm. Butylated hydroxytoluene (BHT) and butylated hydroxyanisole (BHA) served as reference standards. The percentage of radical inhibition was calculated using the previously described Equation (1).

#### 2.4.3 Galvinoxyl Radical (GOR) Scavenging Assay

The free radical scavenging activity of the aqueous extract against galvinoxyl radicals (GOR) was evaluated following the method described by Shi et al. [24]. Briefly, 40 µL of the extract at various concentrations was dispensed into a 96‐well microplate, followed by the addition of 160 µL of the galvinoxyl radical solution (0.1 mM). After an incubation of 2 h, the absorbance was recorded at 428 nm. The percentage of radical inhibition was calculated using Equation (1), and results were expressed as IC_50_ values. Butylated hydroxyanisole (BHA) and butylated hydroxytoluene (BHT) were used as reference antioxidants.

#### 2.4.4 Cupric Reducing Antioxidant Capacity (CUPRAC) Assay

The copper‐reducing capacity of the extract was assessed using the CUPRAC assay, following the method of Apak et al. [25]. Briefly, 40 µL of extract at various concentrations was mixed with 50 µL of CuCl_2_ (10 mM), 50 µL of neocuproine (7.5 mM), and 60 µL of ammonium acetate (1 M). The reaction mixture was incubated for 1 h, and absorbance was measured at 450 nm. Results were expressed as absorbance, and the concentration required to reach an absorbance of 0.5 (A_0_._5_, µg/mL) was determined from the calibration curve. BHA and BHT were used as reference standards.

#### 2.4.5 Ferric‐Reducing Power

The reducing power of the extract was evaluated following the method of Oktay et al. [26], adapted for a microplate format. Briefly, 10 µL of the extract was combined with 40 µL of phosphate buffer (0.2 M, pH 6.6) and 50 µL of potassium ferricyanide [K_3_Fe(CN)_6_, 1%]. The mixture was incubated at 50°C for 20 min, after which 50 µL of trichloroacetic acid (10%), 40 µL of distilled water, and 10 µL of FeCl_3_ (0.1%) were added. The resulting blue–green color was measured at 700 nm. BHA and BHT were used as positive controls. Results were expressed as *A*_0_._5_.

#### 2.4.6 *O*‐Phenanthroline Assay

The ferric ion–reducing activity of the extract was evaluated using the *o*‐phenanthroline assay, following the method of Szydlowska–Czerniaka [27]. In a microplate well, 30 µL of *o*‐phenanthroline (0.5% in methanol), 50 µL of FeCl_3_ (0.2% in distilled water), 110 µL of methanol, and 10 µL of the extract were combined and incubated in the dark for 20 min. Absorbance was measured at 510 nm, and results were expressed as *A*_0_._5_ values (µg/mL). BHT and BHA were used as reference standards.

#### 2.4.7 β‐Carotene Bleaching Assay

The β‐carotene bleaching assay was carried out following the method of Marco [28]. The hydrogen peroxide/β‐carotene/linoleic acid emulsion was prepared according to the protocol described by Ramli et al. [29]. Antioxidant activity was assessed by adding 160 µL of this emulsion to 40 µL of the extract. Absorbance was recorded immediately at 470 nm (*t* = 0 min), and the plate was incubated for 2 h at 50°C. A blank containing the β‐carotene/linoleic acid emulsion with methanol in place of the sample was run in parallel. BHA and BHT were used as reference antioxidants. The percentage of inhibition was calculated using the following formula:

$$I\;\left(\%\right)=\left[1-\frac{\left(A_{\left(t=0\right)}-A_{\left(t=120\right)}\right)}{\left(A_{c\left(t=0\right)}-A_{c\left(t=120\right)}\right)}\right]\times 100$$

where *I* (%) is the percentage of inhibition, *A*_(_ *_t_*_ = 0)_: the absorbance of the tested samples at 0 min, *A*_(_ *_t_*_ = 120)_: the absorbance of the tested samples after 120 min of incubation and *A*_C (_ *_t_*_ = 0)_ is the absorbance of the control (methanol without sample) at 0 min. *A*_C (_ *_t_*_ = 120)_: the absorbance of the control at 120 min. BHT and BHA were used as standards.

### 2.5 Antifungal Activity

The antifungal activity of the aqueous extract of *L. stoechas* against two phytopathogenic fungi, *F. oxysporum* and *A. niger*, was evaluated using the agar dilution method on potato dextrose agar (PDA), following the procedure described by Bendjedid et al. [30]. Fungal inocula were standardized by harvesting actively growing mycelial plugs (5 mm in diameter) from 7‐day‐old cultures and adjusting the spore density to approximately (1 × 10^6^ spores/mL). Three extract concentrations (1.25%, 2.5%, and 5%) were prepared in dimethyl sulfoxide (DMSO). The PDA medium was prepared in the laboratory and sterilized. Thereafter, 1 mL of each extract solution was incorporated into 15 mL of molten PDA and poured into Petri dishes. Once solidified, 5 mm diameter mycelia disks were placed at the center of each plate. Each extract concentration was tested in triplicate. DMSO served as a negative control, while amphotericin B was used as a positive control. Plates were incubated for 7 days at 25°C and monitored daily. Mycelial growth was measured by recording the mycelium diameter along two perpendicular axes, and the average growth was calculated. Antifungal activity was expressed as the percentage of growth inhibition relative to the control and calculated using the following formula:

$$Growth\;inhibition\;\%=\left[\frac{\left(d_{c}-d_{t}\right)}{d_{c}}\right]\times 100$$

where *d*_c_ and *d*_t_ represent the colony diameters in the control and treated plates, respectively.

### 2.6 Statistical Analysis

All compound analyses, antioxidant assays, and antifungal tests were performed in triplicate. Data were analyzed using GraphPad Prism 7 and are presented as mean ± standard deviation (SD). Comparisons among groups were conducted using one‐way ANOVA followed by Tukey's test, with significance set at *p* < 0.05.

## 3 Results and Discussion

### 3.1 Phenolics and Flavonoids Contents

The food, pharmaceutical, cosmetic, and agricultural industries can all benefit from polyphenols, a class of secondary metabolites that are widely distributed in the plant kingdom and have been the focus of extensive research for many years due to their numerous and remarkable biological effects. Therefore, TPC and TFC concentrations in the *L. stoechas* leaf aqueous extract were estimated, and the findings are shown in Table 1. As shown, values of 197.23 ± 0.12 µg GAE/mg for TPC and 166.93 ± 1.15 µg QE/mg for TFC were recorded. Previous studies have also shown that water extraction allows for significant recovery of phenolic compounds, with remarkable levels of TPC and TFC [12, 31]. It is important to note that the aqueous extract of *L. stoechas* grown in Algeria has been shown to have greater levels of TPC and TFC [32], while Elrherabi et al. [31] found similar results for *L. stoechas* from Morocco. Furthermore, values of 232.77 mg GAE/g for TPC and 112.43 mg QE/g for TFC were obtained for the methanolic extract of *L. stoechas* from Tunisia [33].Variations in observed outcomes can be ascribed to the impact of certain elements, including geographical and climatic conditions, the stage of plant development, the duration of storage, the particular plant component utilized, and the extraction technique and solvent employed [5, 18].

**TABLE 1:** The total amount of phenolic and flavonoid compounds in *L. stoechas* aqueous extract.

	Total phenolics (µg GAE/mg)	Total flavonoids (µg QE/mg)
Aqueous extract	197.23 ± 0.12^a^	166.93 ± 1.15^b^

*Note*: Means ± SD (*n* = 3) were used to express the values. Different lowercase letters indicate statistically significant differences among treatments at *p* < 0.05 (one‐way ANOVA followed by Tukey's test). Micrograms of quercetin equivalent per milligram of extract are expressed as µg QE/mg. Gallic acid equivalent micrograms per milligram of extract are expressed as µg GAE/mg.

### 3.2 HPLC Analysis

The identification of phenolic compounds in this study was based on retention times and comparison with commercial standards using HPLC analysis. Although this approach is widely used for preliminary qualitative and quantitative characterization, the absence of confirmatory techniques such as HPLC‐DAD or LC–MS represents a limitation of the present work. Future studies using these advanced analytical tools would allow a more precise structural confirmation of the detected phenolic compounds.

**FIGURE 1:**
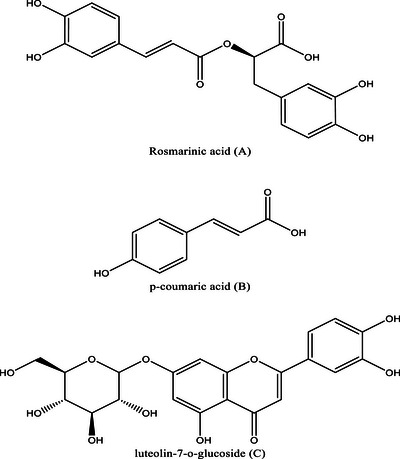
Chemical structures of some major phenolic compounds identified in *L*. stoechas aqueous extract performed using Chemdraw 16.

The HPLC profile of *L. stoechas* aqueous extract is presented in Table 2. The data disclosed the presence of 35 compounds (98.70%) along with other components (1.30%). The polyphenol composition of the extract is primarily represented by phenolic acids (57.5% of the total composition), followed by flavonoids (36.7%), thus forming the major constituents of the extract. Flavonoids are essentially distributed on four main subclasses including flavones (15%), flavonols (10.9%), flavanones (4.2%), and flavanols (1.3%) while the 5.3% were unspecified flavonoids (Figure 1).

**TABLE 2:** Phytochemical composition of *L. stoechas* aqueous extract analyzed by HPLC.

Peaks	RT (min)	Area (%)	Compounds	Formulas	Class/subclass
1	3.67	0.3	Gallic acid	C_7_H_6_O_5_	Phenolic acid
2	4.403	2.7	Protocatechuic acid	C_7_H_6_O_4_	Phenolic acid
3	5.02	1.3	Catechin	C_15_H_14_O_6_	Flavanol
4	7.337	0.3	p‐Hydroxybenzoic acid	C_7_H_6_O_3_	Phenolic acid
5	8.42	0.8	Vanillic acid	C_8_H_8_O_4_	Phenolic acid
6	9.27	0.2	Caffeic acid	C_9_H_8_O_4_	Phenolic acid
7	11.253	1.9	Ferulic acid	C_10_H_10_O_4_	Phenolic acid
8	11.837	2.2	Rutin	C_27_H_30_O_16_	Flavonol
9	13.637	0.9	Sinapic acid	C_11_H_12_O_5_	Phenolic acid
10	14.153	0.4	Quercetin	C_15_H_10_O_7_	Flavonol
11	15.12	1.3	Kaempferol	C_15_H_10_O_6_	Flavonol
12	15.703	0.7	Myricetin	C_15_H_10_O_8_	Flavonol
13	16.603	1.2	Apigenin	C_15_H_10_O_5_	Flavone
14	17.187	0.6	Luteolin	C_15_H_10_O_6_	Flavone
15	17.787	3.6	Naringenin	C_15_H_12_O_5_	Flavanone
16	18.487	0.6	Hesperetin	C_16_H_14_O_6_	Flavanone
17	19.087	1.2	Chlorogenic acid	C_16_H_18_O_9_	Phenolic acid
18	19.753	**25.2**	Rosmarinic acid	C_18_H_16_O_8_	Phenolic acid
19	20.487	2.6	Salvianolic acid (A)	C_26_H_22_O_10_	Phenolic acid
20	21.57	**7.7**	*p*‐Coumaric acid	C_9_H_8_O_3_	Phenolic acid
21	22.653	7.6	Luteolin‐7‐*O*‐glucoside	C_21_H_20_O_11_	Flavone
22	23.503	3.8	Apigenin‐7‐*O*‐glucoside	C_21_H_20_O_10_	Flavone
23	24.053	4.6	Benzoic acid	C_7_H_6_O_2_	Phenolic acid
24	25.053	4.6	Myricetin‐3‐*O*‐glucoside	C_21_H_20_O_13_	Flavonol
25	29.503	4.7	Caffeic acid derivative	Variable	Phenolic acid
26	30.053	2.1	Salvianolic acid (B)	C_36_H_30_O_16_	Phenolic acid
27	32.953	1.0	Luteolin glycoside	C_21_H_20_O_11_	Flavone
28	33.353	1.8	Flavonoid glycoside	Variable	Flavonoid
29	35.437	1.7	Kaempferol derivative	C_15_H_10_O_6_	Flavonol
30	38.47	2.4	Flavonoid dimer	Variable	—
31	39.503	2.6	Caffeic acid oligomer	Variable	Phenolic acid
32	41.27	1.1	Flavonoid glycoside	Variable	—
33	42.37	0.8	Luteolin derivative	C_15_H_10_O_6_	Flavone
34	42.787	2.3	Coumarin derivative	C_9_H_6_O_2_	Coumarin
35	44.37	1.9	Herniarin	C_10_H_8_O_3_	Coumarin
Identified compounds Other components%			98.7% 1.30%		
Total			100%		

*Note*: RT: retention time.

**FIGURE 2:**
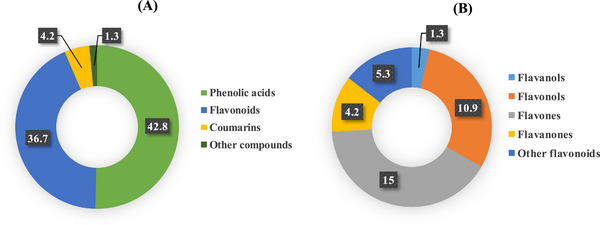
FIGURE 1 The abundance (%) of phenolic compounds found in the aqueous extract of L. stoechas. (A) Distribution of phenolic substances within the main phenolic classes. (B) Distribution of flavonoids within the different subclasses.

Rosmarinic acid (25.2%), *p*‐coumaric acid (7.7%), caffeic acid derivative (4.7%), and benzoic acid (4.6%) were the most abundant phenolic acids while flavonoids were predominately constituted of flavonoid glycosides, including luteolin‐7‐*O*‐glucoside (7.6%), myricetin‐3‐*O*‐glucoside (4.6%), and apigenin‐7‐*O*‐glucoside (3.8%). Naringenin, a non‐glycosylated flavonoid, is one of the compounds that is also present in a notable percentage (3.6%). One of the most important biosynthetic processes for plants in the *Lamiaceae* family is the phenylpropanoid pathway, which produces a variety of phenolic acids, including rosmarinic acid, caffeic acid, and *p*‐coumaric acid [34]. The structure of the predominant compounds identified in the aqueous extract has been illustrated in Figure 2.

Since the extraction process uses water, highly polar phytochemicals are recovered, notably glycosylated ones such as glycosylated flavonoids. Furthermore, hydrophilic phenolic acids, containing several hydroxyl and carboxyl groups, also tend to be soluble in polar solvents and are therefore present in the aqueous extract [35]. Rosmarinic acid constituted also the chief phenolic (80.9%) in the methanolic extract of *L. stoechas* as stated by Karan et al. [36], affirming the present results. Also, phenolic acids including salvianolic acid B (lithospermic acid B, (35%), salvianolic acid derivative (9%), rosmarinic acid (10%), rosmarinic acid isomer (8%), caffeic acid (5%), and flavonoids, such as quercetin 3‑*O*‐glucoside (7%) and apigenin‑O‑glucuronide (6%) formed the major constituents of the aqueous extract of *L. stoechas* [12]. Furthermore, a composition dominated by *p*‐coumaric acid, caffeic acid, myricetin and catechin was reported by Boukada et al. [32]. Recently, Elrherabi et al. [37] identified naringin (38.3%), syringic acid (25.7%) and cinnamic acid (15.9%) as the main constituents of the aqueous extract of *L. stoechas* while 4‐hydroxybenzoic acid, catechin, hydrated catechin and *p*‐coumaric acid were detected at lower levels. The phytochemical profile of plants is subject to variations and is significantly affected by geographical location, climatic conditions, soil quality and altitude, which in turn affects the expression of important enzymes involved in the manufacture of phenolic compounds [38]. Other factors related to experimental procedures, ranging from plant harvesting to subsequent extraction and phytochemical analysis using different chromatographic conditions, also have a significant influence on the results [39, 40, 41, 42].

### 3.3 Antioxidant Activity

The increasing demand from consumers for natural products, which is fueled by worries about the use of synthetic additives or preservatives such as synthetic antioxidants and antimicrobials has spurred numerous studies on plant extracts and their bioactive molecules for possible use in the food sector. These natural substances can extend the shelf life of food by providing protection against oxidation, preventing spoilage, and preserving their nutritional value [43]. Plant extracts can exhibit an antioxidant effect through various mechanisms of action, including the neutralization of free radicals, the termination of the hydrogen subtraction chain in lipids, the chelation of transition metals, and among others. Therefore, in an attempt to determine the antioxidant effectiveness of such mixtures, many techniques have often been employed to thoroughly test the antioxidant capacity [18].

Seven distinct in vitro techniques were used to assess the antioxidant potential of the *L. stoechas* aqueous extract. Methods based on scavenging free radicals (DPPH, ABTS and GOR), the reduction of ions such as ferric iron ions (reducing power and the phenanthroline test) and copper ions (CUPRAC), and the potential to counteract peroxyl radicals in the β‐carotene‐linoleic acid method were used. Table 3 summarizes the antioxidant effect of *L. stoechas* aqueous extract. As can be seen, the extract demonstrated a very strong ability to trap synthetic free radicals DPPH, ABTS and GOR, with respective IC_50_ values of 13.13 ± 0.45, 3.58 ± 0.88 and 8.27 ± 0.56 µg/mL. The antioxidant impact that was seen was similar to the BHA and BHT references. The IC_50_ for BHT in the DPPH test was 22.32 ± 1.19 µg/ml, which is substantially less effective than the extract (*p* < 0.05).

**TABLE 3:** Antioxidant activity of the aqueous extract of *L. stoechas* leaves. .

	DPPH IC_50_(µg/mL)	ABTS IC_50_(µg/mL)	GOR IC_50_(µg/mL)	CUPRAC *A*_0.5_(µg/mL)	Reducing power *A*_0_ (µg/mL)	Phenanthroline *A*_0.5_(µg/mL)	β‐Carotene bleaching IC_50_(µg/mL)
Aqueous extract	13.13 ± 0.45^b^	3.58 ± 0.88^a^	8.27 ± 0.56^a^	3.02 ± 0.06^b^	26.25 ± 1.23^b^	11.79 ± 0.77^a^	52.00 ± 0.95^a^
BHA	5.73 ± 0.41^c^	1.81 ± 0.10^b^	5.38 ± 0.06^b^	3.64 ± 0.19^b^	8.41 ± 0.67^c^	0.93 ± 0.07^c^	1.05 ± 0.03^b^
BHT	22.32 ± 1.19^a^	1.29 ± 0.30^b^	3.32 ± 0.18^c^	9.62 ± 0.87^a^	53.65 ± 0.32^a^	2.24 ± 0.17^b^	0.91 ± 0.01^b^

*Note*: Values are presented as mean ± SD (*n* = 3). Different lowercase letters indicate statistically significant differences among treatments at *p* < 0.05 (one‐way ANOVA followed by Tukey's test). Butyl hydroxyl toluene (BHT) and butyl hydroxylanisole (BHA).

The extract also showed a remarkable ability to decrease copper ions (Cu^2^^+^), with an A_0.5_ value of 3.02 ± 0.06 µg/mL, and was found to be significantly more effective (*p* < 0.05) than the reference BHT (*A*_0.5_ = 9.62 ± 0.87 µg/mL). Concurrently, the extract proved more potent than BHT in reducing ferric ions, using the reducing power test, with respective *A*_0.5_ values of 26.25 ± 1.23 µg/mL and 53.65 ± 0.32 µg/mL. In contrast, when the reduction of ferric ions was assessed using phenanthroline, the extract was found to be significantly less effective (*p* < 0.05) than the two standards, with *A*_0.5_ values of 11.79 ± 0.77, 0.93 ± 0.07, and 2.24 ± 0.17 µg/mL, corresponding, respectively, to the extract, BHA, and BHT.

When an oxidizing agent removes hydrogen atoms from unsaturated fatty acids, like linoleic acid, peroxyl radicals are created. These peroxyl radicals, in turn, attack other fatty acids, leading to a chain reaction. Antioxidants, by their ability to interact with peroxyl radicals and donate hydrogen atoms, can act as termination agents in this chain reaction, thus protecting lipids from oxidation. The aqueous extract of *L. stoechas* showed moderate action against lipid peroxidation evidenced by an IC_50_ of 52.00 ± 0.95 µg/mL, which is comparatively greater than those obtained with the standards (1.05 ± 0.03 µg/mL for BHA and 0.91 ± 0.01 µg/mL for BHT).

The current findings are in line with several papers emphasizing the aqueous extract's potent antiradical activity on DPPH and ABTS [31], the methanolic extract [44] and solvent fractions of *L. stoechas* [32, 45]. It seems to be no earlier reports on the antioxidant activity of *L. stoechas* aqueous extract employing GOR and ABTS assays. Previous reports already highlighted the potent antioxidant capacity of the aqueous extract of *L. stoechas* by studying its ability to reduce ferric ions [37]. Furthermore, a potent antioxidant effect has been reported for extracts obtained using organic solvents as well as for fractions [32, 45]. Nevertheless, the CUPRAC and phenanthroline assays used to determine the antioxidant capacity of the *L. stoechas* aqueous extract are not reported in the literature. The aqueous extract of *L. stoechas* hasbeen described elsewhere in terms of their protection against lipid peroxidation. The current study's results were comparable to those of Elrherabi et al. [31] within the same framework. Amira et al. [46] demonstrated the capacity of the methanolic extract of *L. stoechas* to inhibit lipid peroxidation using the TBARS (Thiobarbituric Acid Reactive Substances) test in an antioxidant model akin to the β‐carotene bleaching test, revealing an IC_50_ of 33 µg/mL against an IC_50_ of 5.5 µg/mL with BHT. The “polar paradox” was explained by Frankel et al. [47], who proposed that extracts made with nonpolar solvents have stronger antioxidant qualities in emulsions made with oil in water because they tend to concentrate at the oil‐in‐water interface, shielding lipids from oxidation. In contrast, polar extracts are not as efficient in preserving lipids because they prefer the aqueous phase.

Plant extracts antioxidant properties are frequently ascribed to their phenolic compound concentration and individual polyphenol makeup. The overall phenolic content of polar fractions and their antioxidant activity are directly correlated, according to Boukada et al. [32]. Moreover, Karan et al. [36] and Dobros et al. [48] highlighted the crucial role of phenolic acids, particularly rosmarinic, caffeic, and ferulic acids, as important sources of *Lavandula* species' antioxidant capacity. The high concentration of phenolic chemicals, particularly rosmarinic acid and its derivatives, in the aqueous extract of *L. stoechas* may be the cause of the exceptional activity seen in it.

It is also worth noting that the pronounced antioxidant activity of the aqueous extract of *L. stoechas* can be attributed to the structural characteristics of its main phenolic constituents, such as the number and location of hydroxyl groups, as well as the presence of substituted molecules, capable of transferring electrons, on the aromatic ring [49]. Rosmarinic acid, the predominant compound, contains two catechol groups (ortho‐dihydroxy groups) on aromatic rings, known for their strong ability to scavenge free radicals. These catecholic structures enhance hydrogen atom donation and facilitate electron transfer, while enabling resonance stabilization of the resulting phenoxyl radicals [50, 51]. This feature supports its ability to neutralize reactive oxygen species such as superoxide and hydroxyl radicals. Rosmarinic acid is reported to act through several complementary mechanisms, including hydrogen atom transfer, single electron transfer, and chelation of transition metals. Metal chelation may limit Fenton‐type reactions and thereby reduce the propagation of lipid peroxidation [52, 53]. In addition to direct radical scavenging, previous studies suggest that rosmarinic acid can influence intracellular antioxidant defenses by modulating enzymes such as superoxide dismutase, catalase, and glutathione peroxidase [54].

Luteolin‐7‐*O*‐glucoside, the main flavonoid, could also contribute significantly to the antioxidant potential of the extract. The flavone backbone possesses a conjugated π‐electron system and a catechol group on the B‐ring (3′,4′‐dihydroxy configuration), which enhances electron delocalization and radical stabilization [55]. Although glycosylation at the C7 position generally reduces antioxidant activity compared to the aglycone (luteolin) due to steric effects and decreased free hydroxyl availability, the glycoside form still retains substantial redox capacity. Furthermore, under certain biological or environmental conditions, hydrolysis of the glycosidic bond may release the more lipophilic and potentially more active aglycone, thereby enhancing antioxidant effectiveness [56].

In contrast, *p*‐coumaric acid which contains only one hydroxyl group on the aromatic ring exhibits comparatively lower intrinsic radical‐scavenging potential. However, its conjugated double bond system (─CH═CH─COOH) contributes to electron delocalization, supporting moderate antioxidant activity [56]. Caffeic acid, another hydrocinnamic acid, contains an ortho‐dihydroxyl group (catechol) on the aromatic ring, recognized as a key determinant of its strong antiradical activity. The presence of adjacent hydroxyl groups enhances hydrogen atom donation and facilitates stabilization of the resulting phenoxyl radical through intramolecular hydrogen bonding and resonance delocalization. Additionally, the conjugated side chain (─CH═CH─COOH) further extends electron delocalization across the molecule, improving its single‐electron transfer (SET) potential. Compared to monophenolic acids such as *p*‐coumaric acid, caffeic acid generally exhibits higher antioxidant efficiency due to this catechol configuration. Therefore, even if present in lower amounts than rosmarinic acid, caffeic acid may significantly contribute to the overall radical‐scavenging and reducing power activities observed [50, 56]. Besides, synergistic interactions between phenolic acids have been reported, where minor constituents regenerate oxidized major antioxidants, prolonging their activity [57] may contribute to the overall antioxidant capacity.

Overall, the strong antioxidant potential of the extract can be mechanistically explained by the abundance of hydroxylated phenolic structures, conjugated systems and catechol groups, which collectively enhance hydrogen atom transfer (HAT) and single‐electron transfer (SET) mechanisms.

### 3.4 Antifungal Activity

**FIGURE 3:**
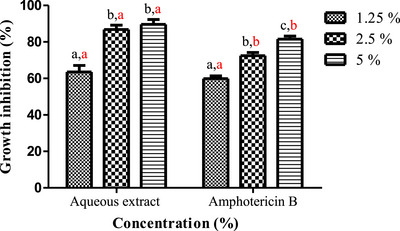
The percentage of *F. oxysporum* growth inhibition caused by aqueous extract concentrations of *L. stoechas* leaves. Values are presented as mean ± SD (*n* = 3). Significant differences among concentrations within each sample are indicated by different lowercase black letters, while significant differences among samples within each concentration are indicated by different lowercase red letters (*p* < 0.05, one‐way ANOVA followed by Tukey's test).

**FIGURE 4:**
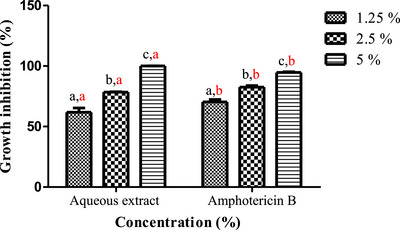
Growth inhibition percentage of aqueous extract concentrations of *L. stoechas*leaves against *A. niger*. Values are presented as mean ± SD (*n* = 3). Significant differences among concentrations within each sample are indicated by different lowercase black letters, while significant differences among samples within each concentration are indicated by different lowercase red letters (*p* < 0.05, one‐way ANOVA followed by Tukey's test).

**FIGURE 5:**
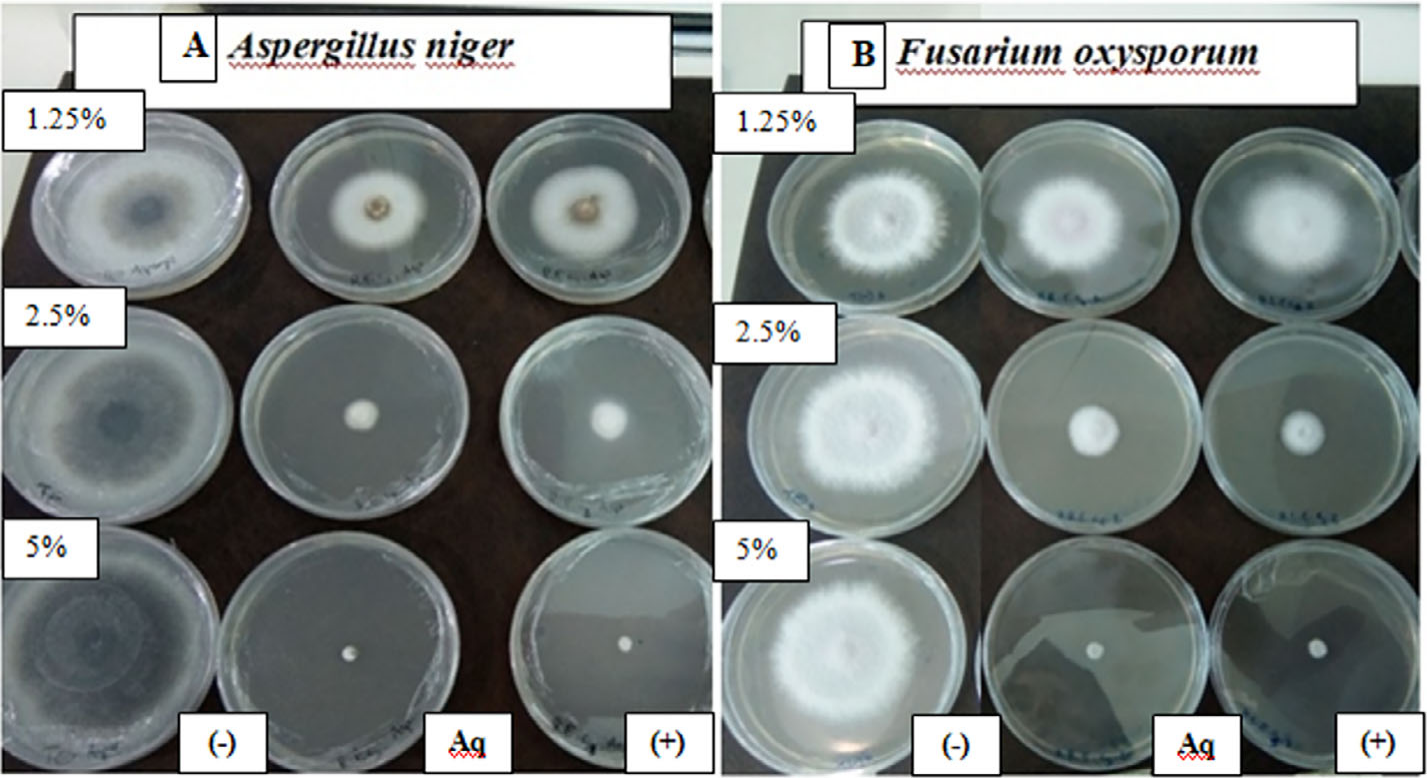
(A) Colonies of *A. niger* and (B) *F. oxysporum* produced on PDA medium supplemented with *L. stoechas* aqueous extract were examined at 5%, 2.5%, and 1.25 percent after 144 h of incubation at 20°C. Negative control (−): untreated control (PDA + DMSO); aqueous extract (Aq); amphotericin B (+).

Plant‐based extracts can constitute safe substitutes and effective alternatives to chemical fungicides for controlling fungal infections after harvest [58]. *A. niger* and *F. oxysporum* known to produce significant post‐harvest financial losses in diverse crops and are also capable of synthesizing toxins that can be metabolized by the human body, thus producing highly toxic derivatives that threaten the health of consumers. The aqueous extract of *L. stoechas* showed very potent inhibitory activity against the tested mycelial strains, in a dose‐dependent manner (Figures 3 and 4). The photographs in Figure 5 illustrate the progressive reduction in the mycelial growth diameter of the two fungal species in media containing different concentrations of the aqueous extract. For *A. niger*, the extract significantly inhibited radial growth at 1.25% and 2.5%, with inhibition rates of 61.76% ± 3.56 and 78.43% ± 0.23, respectively (Figure 5A). Complete inhibition (100%) was observed at the 5% concentration. Similarly, a marked inhibitory effect was observed against *F. oxysporum*. Inhibition rates of 63.54% ± 3.56%, 86.76% ± 2.43%, and 89.62% ± 2.64% were recorded at the extract concentrations of 1.25%, 2.5%, and 5%, respectively (Figure 5B). These results demonstrated a dose‐dependent antifungal effect of the aqueous extract under the tested in vitro conditions.

These results could constitute a first report on the antifungal property of the aqueous extract of *L. stoechas* toward *A. niger* and *F. oxysporum* which could thus be considered as a valid and ecological alternative for industries to combat the undesirable effects linked to fungal proliferation. The antifungal ability of *L. stoechas* is most often described for its essential oil. For instance, Angioni et al. [10] demonstrated that the essential oil derived from various plant parts was highly effective against *F. oxysporum* and *Rhizoctonia solani*, but failed against *Aspergillus flavus*. However, Özcan et al. [59] found that the essential oil had varying effects against *Botrytis cinerea*, *F. oxysporum*, and *Alternaria alternata*. There is strong evidence that the primary phenolic and flavonoid components of plant extracts are responsible for their antifungal qualities [58, 60]. The exact mechanism of action of these substances is still unknown. However, it is assumed that their lipophilic nature, molecular size, and the existence of hydroxyl substituents and other functional groups as well as the occurrence and location of glycosylation, permit them to interfere with biological membrane activities [17, 58]. The antifungal effects of polyphenolic compounds have actually been explained by a number of mechanisms, such as inhibition of glycan and chitin biosynthesis, which causes intracellular components to leak; interference with nucleic acid metabolism through inhibition of mitochondrial processes; and inhibition of important metabolic enzymes [17]. Additionally, Morales et al. [60] suggested that *p*‐coumaric acid's role as an uncoupler of oxidative phosphorylation may be responsible for its suppression of *B. cinerea*.

The aqueous extract of *L. stoechas* exhibited a diverse composition of phenolic compounds that can exert an antifungal effect via different mechanisms of action. It is crucial to emphasize that the most prevalent molecules in the *L. stoechas* aqueous extract are not always the most biologically active; instead, less prevalent components may be more important for the antifungal qualities. The antifungal activity demonstrated by the aqueous extract in the present study likely reflects the combined effects of rosmarinic acid and associated phenolic compounds. Rosmarinic acid, characterized by its catechol structure, has been reported to interact with fungal membranes, particularly those containing ergosterol. Such interactions may alter membrane organization and permeability, leading to leakage of intracellular components and impairment of cellular integrity [61]. Membrane destabilization therefore represents a plausible mechanism underlying the growth inhibition observed. In addition to membrane effects, phenolic compounds may contribute to intracellular oxidative stress in fungal cells. Although rosmarinic acid is widely described as an antioxidant in mammalian systems, phenolics can exert pro‐oxidant effects in microbial contexts depending on concentration and environmental conditions. The resulting accumulation of reactive oxygen species may exceed the detoxification capacity of fungal antioxidant systems, causing damage to proteins, lipids, and nucleic acids [62]. Other phenolics identified in the extract may also reinforce these actions. For example, caffeic acid derivatives have been associated with both membrane perturbation and enhanced oxidative stress in fungal cells [63]. Similarly, *p*‐coumaric acid has also been reported to interfere with cell wall synthesis and spore germination, which could further limit fungal proliferation [64]. Overall, the antifungal activity observed here appears to be multifactorial, involving membrane disruption, redox imbalance, and interference with essential structural processes. The interaction between major and minor phenolic constituents likely enhances the biological effect, supporting a cooperative mode of action rather than a single‐compound mechanism.

### 3.5 Potential Connections Between Antioxidant and Antifungal Properties

Antioxidants can act as free radical scavengers, chain peroxidation breakers, reducing agents, or metal chelators [65]. The structural elements that ensure electron donation and the redox cycle, which underlie antioxidant capacity, can also influence interactions with fungal cells. Specifically, Gawad et al. [66] stated that rosmarinic acid can contribute to essential metal ions chelation in the fungal microenvironment, potentially depriving pathogens of essential micronutrients needed for enzyme function and cellular homeostasis. Additionally, phenolic compounds have been shown to interact with fungal enzymes directly, where hydrogen bonding and aromatic stacking may inhibit key metabolic pathways. Such mechanisms have been proposed for related compounds like caffeic acid and flavonoid aglycones, where antioxidant features also contribute to membrane destabilization or interference with fungal respiration and energy metabolism [67]. Studies in soybean phenolics found correlations between antioxidant activity and antifungal inhibition of mycotoxin producers, implicating specific phenolic acids in dual roles [68].

Thus, the aqueous extract of *L. stoechas* could exert an antifungal effect, probably through its ability to interact with the cellular components of fungi and also through its power to sequester certain metals essential to the growth of these microorganisms. Therefore, further studies should be conducted in this direction in order to establish the mechanism involved in the relationship between antioxidant and antifungal effects.

## 4 Conclusion

The current study highlighted the effective antioxidant and antifungal effects of *L. stoechas* aqueous extract against *A. niger* and *F. oxysporum* as the main causal agents in plant diseases and crop spoilage. These observations suggest the possible application of the extract as a natural substitute for artificial preservatives and agrochemicals. Another benefit is that bioactive substances can be extracted using water rather than hazardous organic solvents, which reduces their environmental impact. Therefore, the food sector can employ the aqueous extract of *L. stoechas* as a safe and eco‐friendly substitute that protects against crop damage and food spoiling, increasing shelf life and guaranteeing food safety while also protecting the environment. However, to fully demonstrate its effectiveness, further studies should be conducted on a broad number of other spoilage agents such as *Botrytis cinerea, Penicillium digitatum*. Besides, the extract must be formulated as an oil‐in‐water emulsion to ensure its effective integration into fats that are highly susceptible to oxidation. Furthermore, bipolymer‐based films incorporating the extract could represent a better approach for preserving fruits and vegetables against fungal infections. Finally, regarding its use in crop protection, its application to seeds is a promising approach for preventing or limiting plant infections during growth.

## Author Contributions

Rima Yakoubi and Samira Bendjedid: conceptualization. Rima Yakoubi and Boubrik Fairouz: methodology. Rima Yakoubi and Namoune Imane: software. Namoune Imane, Boubrik Fairouz, and Lynda Gali: validation. Tarek H. Taha and Anis Ahmad Chaudhary: formal analysis. Tahar Hadj Sadok: investigation. Rima Yakoubi, Namoune Imane, and Boubrik Fairouz: resources. Namoune Imane and Terriche Salim: data curation. Rima Yakoubi, Namoune Imane, Lynda Gali, and Boubrik Fairouz: writing – original draft preparation. Stefania Garzoli, Hamdi Bendif, Walid Elfalleh, and Lynda Gali: writing – review and editing. Djamila Benouchenne: visualization. Stefania Garzoli and Hamdi Bendif: supervision. Hamdi Bendif and Anis Ahmad Chaudhary: project administration. Tarek H. Taha and Walid Elfalleh: funding acquisition. All authors have read and agreed to the published version of the manuscript.

## Funding

This research did not receive any specific grant from funding agencies in the public, commercial, or not‐for‐profit sectors.

## Conflicts of Interest

The authors declare no conflicts of interest.

## Data Availability

The data that support the findings of this study are available from the corresponding author upon reasonable request.
